# Supplementary material for: Broad geographic sampling reveals the shared basis and environmental correlates of seasonal adaptation in Drosophila
Source: eLife. 2021 Jun 22;10:e67577. doi: 10.7554/eLife.67577 (PMC8248982; doi:10.7554/eLife.67577)
Supplement: Transparent reporting form [file elife-67577-transrepform.docx]

***eLife’s* transparent reporting form**

We encourage authors to provide detailed information *within their submission* to facilitate the interpretation and replication of experiments. Authors can upload supporting documentation to indicate the use of appropriate reporting guidelines for health-related research (see [EQUATOR Network](about:blank)), life science research (see the BioSharing Information Resource), or the [ARRIVE guidelines](about:blank) for reporting work involving animal research. Where applicable, authors should refer to any relevant reporting standards documents in this form.

If you have any questions, please consult our Journal Policies and/or contact us: [editorial@elifesciences.org](about:blank).

**Sample-size estimation**

- You should state whether an appropriate sample size was computed when the study was being designed
- You should state the statistical method of sample size computation and any required assumptions
- If no explicit power analysis was used, you should describe how you decided what sample (replicate) size (number) to use

Please outline where this information can be found within the submission (e.g., sections or figure legends), or explain why this information doesn’t apply to your submission:

- Statistical method of effective sample size estimation is in the Materials and Methods.

- The number of populations sequenced was limited by the extent of the DrosRTEC collection effort. The information about these collections is found in the Materials and Methods and Supplementary Table 1.

**Replicates**

- You should report how often each experiment was performed
- You should include a definition of biological versus technical replication
- The data obtained should be provided and sufficient information should be provided to indicate the number of independent biological and/or technical replicates
- If you encountered any outliers, you should describe how these were handled
- Criteria for exclusion/inclusion of data should be clearly stated
- High-throughput sequence data should be uploaded before submission, with a private link for reviewers provided (these are available from both GEO and ArrayExpress)

Please outline where this information can be found within the submission (e.g., sections or figure legends), or explain why this information doesn’t apply to your submission:

- Complete sample size information for collections and sequencing is provided Supplemental Table 1 (referenced in Materials and Methods and Results).

- As our samples are genetic data from wild-caught individuals, technical replication is not relevant.

- Filtering of genetic variants included in the analyses is explained in the Materials and Methods.

- The sequence data accession number is found in Materials and Methods (all sequence data are uploaded to the NCBI SRA database BioProject accession #PRJNA308584). Accession numbers for each sample can be found in Supplemental Table 1.

- VCF files with the raw allele frequencies per population and a R-data file of allele frequencies (compatible with scripts) are available on DataDryad ([https://datadryad.org/stash/dataset/doi:10.5061/dryad.4r7b826](about:blank)), as referenced in the Materials and Methods.

**Statistical reporting**

- Statistical analysis methods should be described and justified
- Raw data should be presented in figures whenever informative to do so (typically when N per group is less than 10)
- For each experiment, you should identify the statistical tests used, exact values of N, definitions of center, methods of multiple test correction, and dispersion and precision measures (e.g., mean, median, SD, SEM, confidence intervals; and, for the major substantive results, a measure of effect size (e.g., Pearson's r, Cohen's d)
- Report exact p-values wherever possible alongside the summary statistics and 95% confidence intervals. These should be reported for all key questions and not only when the p-value is less than 0.05.

Please outline where this information can be found within the submission (e.g., sections or figure legends), or explain why this information doesn’t apply to your submission:

- Description of each statistical test is outlined per test in Materials and Methods.

- The p-values and beta values for all per-SNP tests for seasonality are available at https://github.com/machadoheather/dmel_seasonal_RTEC (as stated in the Data availability section).

- Exact p-values for each test performed (with exclusion of the per-SNP tests above) are found in the Results.

(For large datasets, or papers with a very large number of statistical tests, you may upload a single table file with tests, Ns, etc., with reference to sections in the manuscript.)

**Group allocation**

- Indicate how samples were allocated into experimental groups (in the case of clinical studies, please specify allocation to treatment method); if randomization was used, please also state if restricted randomization was applied
- Indicate if masking was used during group allocation, data collection and/or data analysis

Please outline where this information can be found within the submission (e.g., sections or figure legends), or explain why this information doesn’t apply to your submission:

This does not apply to our submission, as samples are pre-defined as spring/fall and by geographic location. However, our study does perform an analysis of the spring/fall assumption via the 1) permutation tests and the 2) cross-validation analysis (Results and Discussion).

**Additional data files (“source data”)**

- We encourage you to upload relevant additional data files, such as numerical data that are represented as a graph in a figure, or as a summary table
- Where provided, these should be in the most useful format, and they can be uploaded as “Source data” files linked to a main figure or table
- Include model definition files including the full list of parameters used
- Include code used for data analysis (e.g., R, MatLab)
- Avoid stating that data files are “available upon request”

Please indicate the figures or tables for which source data files have been provided:

Code to conduct all analyses and code to reproduce the figures are available at https://github.com/machadoheather/dmel_seasonal_RTEC.
